# Volunteering in adaptive sport: effects of altruistic and egoistic motivation on performance and sustained intention

**DOI:** 10.3389/fspor.2025.1592202

**Published:** 2025-05-08

**Authors:** Keunsu Han, Kyoung Tae Kim

**Affiliations:** ^1^ Department of Kinesiology, Towson University, Towson, MD, United States; ^2^ Department of Health and Human Performance, Kean University, Union, NJ, United States

**Keywords:** sport volunteerism, adaptive sport, volunteer motivation, in-role performance, sustained intention, volunteer management

## Abstract

**Introduction:**

Adaptive sport events provide a unique volunteer setting emphasizing inclusion, accessibility, and individualized support for athletes with disabilities. Volunteers are essential not only to event operations but also to fostering a positive and empowering experience for athletes. Despite extensive research on sport volunteerism, studies focusing specifically on the adaptive sport context remain limited. This study explores how altruistic and egoistic motivations influence volunteers' in-role performance and their sustained intention in adaptive sport settings.

**Methods:**

A self-reported questionnaire was administered to volunteers at the 2024 Korean-American Adaptive Sport Festival in Maryland, USA, using a convenience sampling method. A total of 212 valid responses were included in the analysis. To address the study's objectives, data were analyzed using frequency and descriptive statistics, correlation analysis, exploratory factor analysis (EFA), reliability testing, and multiple regression analysis.

**Results:**

The results indicate that Organization Attachment (OA), Volunteer Attachment (VA), and Internal Benefits (IB) had a positive effect on volunteers' in-role performance, while External Benefits (EB) showed no significant impact. Furthermore, VA and IB had a significant impact on volunteers' sustained intention to continue participating, whereas OA and EB showed no significant effect.

**Discussion:**

These findings offer valuable insights into volunteer motivation and its impact on in-role performance and continued engagement in adaptive sport settings. The study offers practical implications for building a more committed, capable, and sustainable volunteer workforce to support the continued success of adaptive sport programs.

## 1 Introduction

Adaptive sports are essential in fostering inclusivity, empowerment, and social integration for individuals with disabilities (1). These sports are specifically adapted to accommodate a range of physical, intellectual, or visual impairments allowing athletes to improve their physical health, build self-confidence, and engage in both competitive and recreational activities (2–4). Major adaptive sporting events, such as the Paralympics and regional adaptive sport festivals, serve as key platforms not only for showcasing the abilities and achievements of athletes with disabilities but also for promoting disability rights, social inclusion, and equal opportunities in athletics (5, 6). In addition to benefiting athletes, these events depend heavily on volunteers, who play a pivotal role in managing event logistics, assisting athletes, and fostering an inclusive and supportive environment (7).

Although the importance of volunteer service in sporting events has long been recognized, significant challenges in recruiting and retaining volunteers still persist (8–11). Ensuring that volunteers remain engaged and perform their roles effectively is critical to the sustained success of adaptive sport programs. Therefore, identifying and understanding the motivational factors that drive volunteers to contribute and continue their involvement in these events is essential for maintaining a strong and committed volunteer workforce.

Volunteers are a cornerstone of sporting events, providing essential support in logistics, athlete services, and event management (12–15). While extensive research has explored volunteer motivation in mainstream sports, there is a growing need to examine how these motivations function in adaptive sports. These experiences do not inherently suggest that volunteering in adaptive sports is more difficult or altruistic. Rather, they emphasize the importance of contextual factors such as the nature of the athlete-volunteer relationship and considerations around accessibility, which may influence how volunteers approach and engage with their roles. Gaining a deeper understanding of these nuanced dynamics is essential for examining how motivational factors shape volunteer performance and retention in adaptive sport settings (16).

The study of volunteer motivation has evolved significantly over the past few decades, moving from early satisfaction-based models to more comprehensive frameworks that account for a range of psychological, social, and contextual influences (17). Foundational work such as the functional approach proposed by Clary et al. (18) emphasizes that individuals engage in volunteering to fulfill specific psychological functions, including values, understanding, social interaction, career advancement, and personal enhancement. Similarly, self-determination theory (19) distinguishes between intrinsic and extrinsic motivations, highlighting the importance of autonomy, competence, and relatedness in sustained volunteer engagement. Additional perspectives, such as role identity theory (20), suggest that volunteers who internalize their roles as part of their self-concept are more likely to exhibit long-term commitment and consistent performance.

This growing body of research has established strong links between motivational factors and important volunteer outcomes, including in-role performance—the effective completion of assigned tasks (21)—and continuance intention, or the desire to volunteer again in the future (22, 23). While these theories have been widely applied in diverse volunteer settings such as health care, education, and mega-sporting events (8, 63), limited attention has been given to how they function in the context of adaptive sports.

Adaptive sport events represent a unique volunteer environment due to their emphasis on inclusion, accessibility, and individualized athlete support. Volunteers in these settings not only contribute to logistical and operational tasks but also help foster empowering and socially supportive experiences for athletes with disabilities. Understanding how motivational factors influence volunteer outcomes in this context is therefore essential. However, empirical studies that examine volunteer motivation specific to adaptive sports remain scarce, leaving unanswered questions about which motivational dimensions best predict both immediate performance and long-term engagement. This study seeks to address this gap by situating adaptive sport volunteering within the broader landscape of volunteer motivation research and by testing established theoretical models in a novel and underexplored context.

To address this gap, the present study examines how four key motivational factors—organization attachment, volunteer attachment, internal benefits, and external benefits—influence volunteers’ in-role performance and intention to continue volunteering at an adaptive sport festival. Conducted at the 2024 Korean-American Adaptive Sport Festival, this study seeks to uncover which motivational constructs enhance or diminish both volunteer effectiveness and long-term commitment. Findings from this research will contribute to the broader literature on volunteer motivation and provide practical insights for adaptive sport organizations, helping them refine recruitment, training, and retention strategies. By fostering a stronger and more committed volunteer base, adaptive sport programs can continue to expand their reach, enhance event quality, and further promote equity and inclusion in sports.

## 2 Review of literature

### 2.1 Theoretical frameworks

The study of motivation has long been one of the most popular topics in fields such as management, organizational behavior, and human resource management, and the study of sport volunteers is no exception. It is widely recognized that volunteers play a crucial role in the success of sporting events at all levels, from mega-events to local, regional, professional, amateur, and community-based competitions. For decades, research on volunteer motivation has remained a key focus in understanding their engagement and contribution.

Various theoretical frameworks have been used in research on motivation, including Equity Theory (24), Expectancy Theory (25) and Herzberg's Motivation-Hygiene Theory (26). In the context of understanding volunteer motivation in adaptive sports, need-based theories such as Maslow's Hierarchy of Needs, the ERG (Existence, Relatedness, Growth) Theory, and Self-Determination Theory (SDT) (27) provide a well-conceptualized framework. Maslow (28) proposed a five-tiered model of human needs, arranged in a hierarchical structure: physiological, safety, love/belonging, esteem, and self-actualization. According to Maslow, individuals must satisfy lower-level needs before progressing to higher-level ones. In the context of volunteer motivation, this theory suggests that volunteers may initially seek to fulfil basic needs, such as social belonging (e.g., forming relationships with others in the organization) or esteem (e.g., gaining recognition for their contributions). As volunteers become more engaged, they may strive for self-actualization by finding deeper meaning and personal fulfilment through their volunteer work (8, 18). Research has shown that sport volunteers are often driven by social connection, personal development, and the desire to contribute to a cause they find meaningful (15).

Alderfer (29) refined Maslow's model into three broad categories: Existence, Relatedness, and Growth (ERG). Unlike Maslow's strict hierarchy, ERG Theory allows for flexibility, acknowledging that individuals may pursue different needs simultaneously. Existence needs encompass basic survival necessities, such as food, shelter, and financial security. For sport volunteers, this could relate to compensation in terms of travel reimbursement, food provisions, or appropriate working conditions. Relatedness needs involve meaningful relationships and social interactions, as volunteers often seek to build connections with fellow volunteers, athletes, and event organizers (13, 14, 30). Growth needs focus on personal development and self-improvement, motivating volunteers through opportunities to learn new skills, gain leadership experience, or enhance their professional profile (31). ERG Theory also introduces the concept of frustration-regression, meaning that if higher-level needs, such as growth, are unmet, individuals may return to focusing on lower-level needs, such as relatedness or existence. This flexibility makes ERG Theory particularly relevant for adaptive sports volunteers, whose motivations may shift based on personal experiences and the organizational environment (8, 10, 11).

Deci and Ryan (19) developed Self-Determination Theory (SDT) as a framework for understanding motivation based on the extent to which behaviors are self-directed and fulfilling. SDT distinguishes between intrinsic motivation, where individuals engage in activities for inherent satisfaction, and extrinsic motivation, where behaviors are driven by external rewards or pressures. The theory posits that individuals have three fundamental psychological needs: autonomy, competence, and relatedness. Autonomy refers to the need for self-direction and control over one's actions. In the context of sport volunteering, individuals who perceive their roles as self-chosen rather than imposed are more likely to experience higher motivation and satisfaction (32). Competence involves the need to develop skills and feel effective in one's environment. Volunteers often seek opportunities to learn new skills or apply their existing expertise, enhancing their sense of accomplishment. Relatedness reflects the need for meaningful social connections (27, 30). Many volunteers are motivated by the chance to interact with athletes, coaches, and other volunteers, fostering a sense of belonging and shared purpose (33). SDT has been widely applied in sports and volunteerism research, showing that volunteers who experience autonomy, competence, and relatedness tend to have higher engagement, longer retention, and greater overall satisfaction (8, 34, 35). The integration of Maslow's Hierarchy of Needs, ERG Theory, and SDT provides a comprehensive framework for understanding why individuals choose to volunteer in sport events. While some volunteers may initially engage due to relatedness needs (e.g., social connection), others may be driven by competence and growth (e.g., skill development and leadership opportunities). By applying these motivational theories, organizations can develop targeted recruitment and retention strategies to ensure sustained volunteer engagement in adaptive sports programs.

In addition to these theoretical perspectives, a growing body of research has explored the experiences of volunteers at parasport events, offering valuable contextual insights—even when not explicitly centered on motivational constructs. Notably, the work of Dickson and colleagues provides a nuanced understanding of the social impact and legacy associated with volunteering in these unique settings. For instance, Dickson, Terwiel, and Vetitnev (65) documented the emergence of a social legacy from volunteer participation at the Sochi 2014 Olympic and Paralympic Winter Games, illustrating how such experiences can foster long-term civic engagement and community attachment. Similarly, earlier research on the Vancouver 2010 Games highlighted how event-specific identity and a shared sense of purpose shaped the volunteer experience (66).

Beyond the exploration of legacy outcomes, several studies have examined inclusive volunteering practices and their implications. Dickson, Darcy, and Benson (67) investigated the experiences of volunteers with disabilities at the London 2012 Olympic and Paralympic Games, focusing on who participated, their motivations, and their intentions to volunteer again. This study sheds light on the diversity of the volunteer base and underscores the significance of inclusive event design in promoting positive volunteer outcomes. Further, Dickson, Darcy, and Walker (68) examined how parasport events can be strategically leveraged to generate sport participation and tourism legacies. Their longitudinal case study of the Whistler Adaptive Sports Program illustrates how targeted initiatives introduced during mega-events can deliver lasting benefits for adaptive sport programs and broader community engagement.

While these studies have made significant contributions to understanding parasport volunteerism, much of the literature remains focused on volunteers at large-scale or mega-events. As a result, relatively little is known about volunteer motivation and experience in community-based adaptive sport settings, which often rely heavily on episodic volunteers and local engagement. Compared to the structured, resource-rich environments of mega-events, community-based adaptive sport festivals may present different organizational challenges and relational dynamics. This study addresses this gap by focusing specifically on motivational factors and volunteer outcomes within a community-based adaptive sport context, contributing new insights into how volunteers engage with and sustain their involvement in grassroots-level parasport initiatives.

### 2.2 Volunteer motivation

Understanding the key constructs that drive sport volunteer motivation is crucial for developing effective volunteer management strategies. Research has identified several fundamental motivational constructs, which can be broadly categorized into altruistic motives, social motives, personal development motives, extrinsic rewards, and psychological fulfillment (13, 14, 18, 36).

Farrell et al. (37) explored volunteer satisfaction and motivation by developing the Special Event Volunteer Motivation Scale (SEVMS), a 28-item instrument that identifies four motivational dimensions: purposive (contributing to the community and event), solidary (social interaction, group identification, and networking), external tradition (family tradition, use of free time, and career development), and commitment (alignment between personal expectations and one's skills). Twynam et al. (38) tested the SEVMS model among sport event volunteers, and it has since been applied in various contexts, including international volunteering. The Olympic Volunteer Motivation Scale (OVMS), developed by Giannoulakis (39), has become a foundational instrument in examining volunteer motivations for Olympic and other mega-sport events. The OVMS identifies multiple dimensions—such as values, career, social, understanding, ego enhancement, and patriotism—capturing both intrinsic and extrinsic motivations. Building on this foundation, Bang & Chelladurai (36) developed the Volunteer Motivation Scale for International Sporting Events (VMS-ISE) to capture motivational dimensions specific to global sport contexts. The VMS-ISE includes categories such as expression of values, patriotism, interpersonal contacts, personal growth, career orientation, and extrinsic rewards and love of sports offering insight into motivations shaped by cultural exchange and global engagement. Its validation across diverse event settings makes it particularly valuable for cross-cultural studies of volunteer motivation (40).

Many volunteers are motivated by a sense of social responsibility and the belief that their contributions positively impact athletes and communities. Studies indicate that individuals with strong humanitarian motives experience greater intrinsic satisfaction and are more likely to remain engaged in volunteer work over time (18). The opportunity for social interaction is another critical factor influencing volunteer motivation in sports. Many volunteers seek to build relationships, expand their networks, and feel a sense of belonging within a community (13, 14). Events that foster team dynamics and meaningful interpersonal relationships tend to have higher volunteer retention rates (31). Volunteering in sport also serves as a pathway for personal growth and skill development. Many individuals seek learning opportunities, leadership experience, or a sense of accomplishment through their volunteer work. Research suggests that volunteers are often motivated by the potential to gain transferable skills relevant to their professional or personal lives. For students or young professionals, volunteering can provide hands-on experience that enhances career prospects and professional networks (15, 41, 42).

Although intrinsic motivation is often the primary driver of volunteerism, extrinsic rewards can also play a meaningful role in sustaining volunteer engagement. Incentives such as certificates, public recognition, free event access, travel reimbursement, or modest financial rewards can reinforce a sense of value and appreciation among volunteers (8, 23). While typically secondary to intrinsic motives, these rewards may enhance satisfaction and commitment—particularly for those investing significant time and effort in large-scale sporting events.

In addition to these motivational drivers, volunteering has been associated with a range of psychological benefits. Participation can foster a sense of purpose, emotional satisfaction, and personal accomplishment (35). These effects may be especially pronounced in adaptive sports settings, where volunteers frequently report heightened empathy, resilience, and emotional fulfillment due to the meaningful nature of their interactions. Furthermore, volunteer involvement can act as a coping mechanism for stress, anxiety, or life transitions, further reinforcing the psychological value of sustained engagement (16, 33).

Han et al. (41) enhanced the measurement of sport volunteer motivation by refining the Volunteer Motivation Questionnaire (OVMQ) and introducing a four-factor model that integrates key motivational constructs. This model identifies altruism and egoism as the two primary motives for volunteering, further dividing them into two subdimensions each. Altruistic motivation consists of organization attachment (OA) and volunteer attachment (VA). Organizational attachment reflects a deep sense of pride, loyalty, and commitment to an organization's mission and success. It involves a strong emotional connection that drives volunteers to actively support and advocate for the organization's goals. This attachment is often reinforced by shared values, a sense of belonging, and meaningful experiences within the organization. For Olympic volunteers from the host nation, patriotism frequently serves as a powerful motivator, strengthening their dedication and enthusiasm for their roles (40, 41, 43). Similarly, in the Korean-American Adaptive Sports Festival, which serves as a case for this study, Korean volunteers may be more actively involved due to their national connection and sense of pride. This motivation closely aligns with that of Olympic volunteers, for whom patriotism plays a key role. Beyond national pride, volunteers may also develop a lasting connection to the organization through personal growth, social bonds, and a shared purpose.

Volunteer attachment reflects a deep emotional and psychological connection to one's role, the communities served, and the broader mission of volunteerism. It is driven by a genuine concern for others and society, highlighting the positive impact of volunteering and the goodwill associated with making a meaningful contribution. This attachment often stems from a sense of responsibility, moral obligation, and the belief that one's efforts help create a more compassionate and supportive society. Additionally, volunteer attachment includes a sense of self-pride derived from helping others, reinforcing personal values, and fostering a sense of purpose. Volunteers who feel strongly connected to their work are more likely to remain engaged, experience emotional fulfilment, and develop a long-term commitment to volunteerism. Beyond benefiting those who receive assistance, this attachment strengthens social bonds and enhances the overall well-being of volunteers, creating a more engaged and cohesive community (37, 41).^1^ While prior studies have not explicitly examined motivational processes in adaptive sport volunteering, certain conceptual factors may be particularly relevant in this context. In the context of adaptive sporting events, which serve as the focus of this study, it is proposed that volunteer attachment may be shaped by factors such as empathy, a sense of personal achievement through social contribution, and a deep aspiration to support athletes with disabilities. While these assumptions have not been extensively examined in prior research, they offer a meaningful framework for understanding the potentially unique motivational dynamics in adaptive sports settings. This study seeks to explore whether and how these factors influence volunteer attachment, performance, and sustained engagement within adaptive sport contexts.

Egoistic motivation encompasses both internal benefits (IB) and external benefits (EB). Internal benefits include personal growth, the acquisition of new experiences, networking opportunities, and career development (37, see text footnote 1), with the “Love of Sport” factor (8) also falling within this category. External benefits, on the other hand, involve tangible rewards such as free uniforms, meals, academic credits, fulfillment of school requirements, and event access, which can be particularly appealing to younger or student volunteers (44, 45) The conceptualization of IB and EB remains consistent across previous research in sporting settings.

This refined framework offers a more structured and streamlined approach to understanding sport volunteer motivation, distilling complex traditional motivational factors into a clearer model while still aligning volunteer management strategies with the diverse needs and incentives of volunteers in sporting events. Specifically, in adaptive sports—where athletes often navigate distinct physical and social challenges—understanding how motivational constructs operate may be particularly important. Volunteers in these settings may be influenced by a combination of altruistic and egoistic motives, suggesting the need for organizations to consider both intrinsic and extrinsic drivers of engagement. It is also possible that the personal connections formed between volunteers and adaptive athletes contribute to volunteer attachment, while experiences of accomplishment and social contribution may foster a sense of organizational attachment. By exploring these possibilities, this study aims to inform the development of more effective volunteer recruitment and retention strategies that support long-term commitment, enhance volunteer performance, and contribute to a meaningful volunteer experience in adaptive sport contexts.

Recognizing the blend of altruistic and egoistic motivations in adaptive sport volunteering also invites consideration of broader perspectives from non-event contexts and critical disability studies. In addition to event-based volunteering, research on volunteers in non-event disability sport programs provides further insights into the motivations and meanings behind volunteer engagement. While the contexts differ, common motivations such as altruism, personal interest in sport, and a desire to support marginalized groups often emerge. Some studies suggest that volunteers are drawn to disability sport out of a desire to “give back” or help those perceived as less fortunate (13, 14), but disability studies scholars have cautioned against uncritically framing these motivations. The concept of benevolent ableism describes well-intentioned attitudes that, while seemingly positive, reinforce power imbalances and paternalistic views toward people with disabilities (46). Recognizing these tensions is crucial for interpreting volunteer narratives and avoiding assumptions that frame disability solely in terms of need or deficiency. By incorporating literature from both event and non-event contexts, as well as from critical disability studies, this study offers a more nuanced understanding of the complex drivers behind disability sport volunteering.

Recent scholarship continues to expand our understanding of volunteer motivations in sport contexts, with an increasing focus on regional, demographic, and experiential factors. Bańbuła (47), for example, explored motives for participating in sport event volunteering in Poland and found that personal development, social interaction, and identification with sport were among the most commonly cited motivations. The study also emphasized how volunteers' educational background and prior experience influenced their engagement, suggesting that motivations can shift based on socio-cultural and individual factors. Complementing this, Rozmiarek, Poczta, and Malchrowicz-Mośko (48) examined motivations of volunteers at the 2023 European Games in Poland, identifying a blend of altruistic and self-oriented motives, including the desire to contribute to event success and to gain professional experience. The findings highlight the continuing relevance of both intrinsic and extrinsic motivations, while also pointing to the increasing importance of employability-related incentives, particularly among younger volunteers.

Further advancing this line of research, Rozmiarek et al. (49) investigated the motivations of student volunteers in relation to their professional aspirations and previous volunteer experiences. Their results suggest that motivations are shaped not only by personal values but also by career-oriented goals and accumulated volunteering experience. This underscores the dynamic nature of volunteer motivation over time and across life stages, reinforcing the need for tailored recruitment and retention strategies in sport event volunteer management. Together, these recent studies contribute to a more current and comprehensive view of volunteerism in sport, reinforcing key themes in the literature while bringing new dimensions—such as generational trends and professional development—into sharper focus. Including these contributions strengthens the foundation for analyzing volunteer motivation in both mainstream and adaptive sport event contexts.

Beyond motivations rooted in disability sport and altruism, cultural and ethnic identity can also play a meaningful role in shaping volunteer participation. The event examined in this study attracted a volunteer base with strong ties to the Korean diaspora, suggesting that cultural affinity may be a motivating factor alongside interest in adaptive sport. Although research specifically addressing ethnic or diasporic motivations in sport event volunteering is limited, broader literature on service and community engagement indicates that individuals often volunteer in culturally familiar settings as a way to affirm identity, foster belonging, and maintain connections with heritage communities (50, 51). For diaspora populations in particular, volunteering can serve as a form of civic engagement that bridges cultural pride and communal responsibility (52). These insights are important for understanding the layered motivations at play in contexts where sport, disability, and cultural identity intersect, and they highlight the need for more research on ethno-cultural dimensions of sport event volunteering.

### 2.3 In-role performance of volunteers

In-role performance is often associated with job performance, but the two concepts are not identical. Job performance has long been recognized as a critical determinant of both employee and organizational success. It is a broad concept that encompasses both in-role performance and extra-role behaviors, such as organizational citizenship behavior (OCB) or discretionary efforts. Job performance reflects an employee's overall effectiveness in carrying out tasks and responsibilities that contribute to organizational success (21). Over time, the study of job performance has evolved alongside research on motivation, shifting from early theories primarily focused on job satisfaction to more contemporary perspectives that emphasize employee engagement and job crafting (53).

In-role performance, also known as task performance, refers to an individual's execution of formal duties and responsibilities as outlined in their job description. It involves completing assigned tasks efficiently and effectively without necessarily extending beyond formal job expectations (54). The concept of role performance in organizations dates back to Katz and Kahn (55), who emphasized the significance of role behaviors in maintaining organizational effectiveness. Building on this foundation, Borman and Motowidlo (21) differentiated task performance (in-role performance) from contextual performance, defining in-role performance as behaviors directly tied to fulfilling job responsibilities and contributing to organizational goals. This distinction has been further refined in organizational behavior literature to differentiate required duties (in-role behaviors) from discretionary, extra-role behaviors, such as organizational citizenship behavior (OCB). In this study, in-role performance is particularly relevant, as most volunteers were episodic participants in a two-day event. Their primary focus was on fulfilling assigned responsibilities to enhance the overall event experience and support athletes with disabilities.

Motivation has been a critical factor in shaping in-role performance, directly impacting how effectively individuals fulfill their assigned responsibilities. Research has consistently shown that highly motivated employees and volunteers are more likely to engage in in-role behaviors, thereby enhancing organizational efficiency and goal attainment (32). Various theoretical frameworks, including self-determination theory (SDT), expectancy theory, and goal-setting theory, offer insights into how motivation drives performance.

Self-determination theory (SDT) (19) distinguishes between intrinsic and extrinsic motivation, both of which impact in-role performance. Intrinsically motivated individuals, who engage in tasks due to personal satisfaction and interest, exhibit higher levels of effort, persistence, and commitment to their roles (27). In contrast, extrinsically motivated individuals, who perform tasks in response to external rewards or pressures, may effectively fulfill their in-role duties but are often less likely to engage in extra-role behaviors that go beyond their assigned responsibilities.

Within the context of sport volunteerism, motivation significantly influences the extent to which volunteers engage in their required tasks. Affective commitment, role clarity, and perceived organizational support have been identified as key motivational factors that enhance in-role performance among volunteers (8, 23, 37). Volunteers who feel emotionally connected to the organization and have a clear understanding of their roles are more likely to perform effectively and sustain their involvement over time.

The relationship between motivation and in-role performance is well-documented across various organizational settings (27, 56). By fostering both intrinsic and extrinsic motivators, organizations can enhance volunteer engagement, ensuring that individuals not only fulfill their assigned responsibilities but also contribute to the broader success of the organization.

### 2.4 Sustained intention to volunteer

The decision to continue volunteering is shaped by a combination of motivational and organizational factors that influence long-term engagement. Sustained intention to volunteer, also referred to as continuance intention, reflects an individual's commitment to ongoing participation based on personal motivations, organizational support, and prior experiences (18, 57). Research suggests that volunteers are more likely to remain engaged when they experience personal fulfillment, a sense of belonging, and alignment with their intrinsic and extrinsic motivations (32).

Organizational support and role clarity significantly impact a volunteer's willingness to continue. Volunteers who feel valued through training, recognition, and meaningful responsibilities tend to remain engaged over time (13, 14, 45). Additionally, leadership, job satisfaction, and affective commitment—an individual's emotional attachment to an organization—play a crucial role in sustaining volunteer participation. Volunteers who find fulfillment in their roles and develop a personal connection to the organization's mission are more likely to remain engaged over the long term (8, 15, 58). Another key factor is psychological contract fulfillment, which refers to the perception that the organization meets the expectations and values of its volunteers. When volunteers feel that their contributions are recognized and appreciated, they are more likely to continue volunteering (22). Role identity theory further suggests that individuals who integrate their volunteer roles into their personal identity exhibit higher levels of sustained participation (20).

Motivation serves as a key determinant of sustained intention, directly influencing a volunteer's commitment to long-term service. Self-determination theory (SDT) (19) explains that intrinsically motivated individuals—those who volunteer out of personal satisfaction, passion, and a sense of purpose—demonstrate greater persistence and long-term engagement than those primarily driven by external rewards (27). While intrinsic motivation plays a central role, extrinsic motivators also contribute to continued participation. Expectancy theory (59) suggests that volunteers are more likely to stay engaged if they believe their efforts will lead to valued outcomes, such as skill development, networking, or social recognition (22). Volunteers who perceive tangible benefits and organizational appreciation exhibit higher levels of commitment (13, 14).

In sport volunteerism, sustained engagement is particularly linked to a sense of community, social interactions, and opportunities for personal growth (31). Organizations that foster clear communication, well-defined roles, and ongoing support help strengthen volunteer motivation and retention. Additionally, fulfilling volunteers' psychological contracts—ensuring their expectations align with their experiences—further enhances continuance intention (22).

Overall, sustained volunteerism is influenced by multiple factors, including intrinsic motivation, extrinsic incentives, job satisfaction, role clarity, leadership, affective commitment, and organizational support. By cultivating a meaningful volunteer experience, organizations can strengthen long-term retention, ensuring that volunteers remain dedicated, motivated, and actively engaged.

## 3 Research methods

### 3.1 Participants

Participants in this study consist of volunteers from the 2024 Korean-American Adaptive Sport Festival in Maryland, a semi-annual national event. The festival features over 1,000 athletes with disabilities, along with their families, competing in 14 different sports, supported by more than 300 volunteers. This study utilizes non-probability sampling, specifically convenience sampling.

A total of 250 surveys were collected both on-site during the two-day event and via email after the festival. Each morning, orientation sessions were held for specific volunteer groups whose roles required additional skills, training, or safety preparation (e.g., assistant referees, media assistants, drivers). Surveys were collected during these orientations. For volunteers who did not complete the questionnaire on-site, the volunteer coordinator agreed to distribute the survey via email after the event to ensure broader participation. Prior to participation, individuals were informed about the study's purpose, and only those who provided voluntary consent completed a self-assessment questionnaire. Following data screening, 38 responses were excluded due to inconsistencies, yielding a final sample of 212 valid questionnaires for analysis.

This study was conducted in accordance with established ethical guidelines to ensure the protection of participants and the integrity of the research process. Specifically, the research adhered to the ethical principles outlined in the *Publication Manual of the American Psychological Association* (7th edition), including informed consent, voluntary participation, confidentiality, and the right to withdraw at any time. The study received approval from the Institutional Review Board (IRB) at Towson University, and all participants provided informed consent prior to their involvement in the study.

### 3.2 Instrumentation

The questionnaire was structured into two main sections. The first section gathered demographic details from event volunteers, including gender, race, marital status, employment status, and prior volunteering experience. The second section focused on measuring three key constructs: volunteer motivation, in-role performance, and sustained intention to volunteer. To ensure the survey aligned with the study's objectives, the variables were adapted from previously validated and reliable measurement scales. A panel of experts assessed the content validity, and the questionnaire was refined based on their recommendations. The final version was designed for self-administration and employed a 5-point Likert scale, ranging from 1 (“Strongly Disagree”) to 5 (“Strongly Agree”).

Volunteer motivation comprises four distinct factors: volunteer attachment (VA), organization attachment (OA), internal benefits (IB), and external benefits (EB), each measured using three items adapted from Han et al. (41). In-role performance was assessed with three items modified from Williams and Anderson (54), while sustained intention to volunteer was evaluated using three items adapted from Clary and Snyder (60), with necessary adjustments. To ensure alignment with the study's objectives and the relevance of the content, all questionnaire items were thoroughly reviewed, refined, and expanded as necessary. The final instrument comprised 18 items, with three items representing each of the six factors: volunteer attachment (VA), organizational attachment (OA), internal benefits (IB), external benefits (EB), in-role performance (IRP), and sustained intention (SI). Construct validity and reliability for this sample were assessed using Exploratory Factor Analysis (EFA) and Cronbach's alpha, with the results presented in Tables 2, 3.

### 3.3 Data analysis

Data analysis for this study was performed using SPSS version 29.0, employing a range of statistical techniques to examine the study variables. First, frequency analysis was conducted to summarize the demographic characteristics of the participants. Second, descriptive statistics were used to compute the means and standard deviations of all study variables. Third, correlation analysis was performed to explore relationships between variables and detect potential multicollinearity issues. Fourth, exploratory factor analysis (EFA) was utilized to identify underlying factors and validate the measurement constructs. Fifth, reliability analysis was conducted using Cronbach's α to assess the internal consistency of each variable's items. Lastly, multiple regression analysis was conducted to evaluate the influence of volunteer motivation on in-role performance and sustained intention to volunteer among participants in an adaptive sport festival. A standard multiple regression using ordinary least squares (OLS) estimation was employed, with all predictors entered simultaneously to assess their unique contributions. The assumptions of multiple regression, including linearity, normality, multicollinearity, and homoscedasticity, were assessed and satisfied prior to analysis, following the guidelines of Hair et al. (61).

## 4 Results

### 4.1 Demographics

The demographic analysis of the sample, based on 212 valid responses, indicated that 60.1% of respondents were female, 39.8% were male, and 0.1% identified as other**.** Regarding marital status**,** nearly 39.8% were single, 56.1% were married, 3.3% were divorced, 1.4% were widowed, and 2.7% fell into other categories**.**

In terms of ethnic composition, the majority of participants were Asian-American (96.7%), reflecting the festival's organization by the Korea ParaSports Association of USA and the predominant involvement of Korean-American athletes, staff, and volunteers. Additionally, 1.3% identified as White/Caucasian, 0.2% as African-American, 1.6% as Hispanic, and 0.2% as other.

For employment status, 57.9% of participants were employed full-time, 21.7% worked part-time, 2.1% were unemployed, 17.6% were retired, and 0.7% fell into other employment categories**.**

Volunteer experience varied among participants, with the largest group (52.2%) having volunteered 4–8 times in general, followed by 17.9% with 1–3 instances, 13.4% with 9–15 instances, 11.7% with 16–25 instances, and 4.8% having volunteered more than 25 times. Additionally, 47% of volunteers reported prior experience with adaptive sport events before participating in this festival.

### 4.2 Analysis of correlation values and descriptive statistics

Table 1 presents the correlation coefficients, means, and standard deviations for all variables used in this study. The correlation values ranged from .218 to.591, all of which were statistically significant. While the variables were correlated, they remained distinct, with all correlation values falling below the ±.85 threshold recommended by Kline (62), indicating the absence of multicollinearity.

**Table 1:** Results of correlation values and descriptive statistics.

Factor	1	2	3	4	5	6	M	SD
1. OA	1						4.37	.959
2. VA	.338*	1					4.76	.816
3. IB	.371*	.289*	1				4.26	.943
4. EB	.239*	.218*	.591*	1			3.63	.810
5. IRP	.297*	.331*	.301*	.298*	1		4.62	.881
6. SI	.351*	.415*	.330*	.263*	.471*	1	4.31	.807

* *p* < .01.

OA, organizational attachment; VA, volunteer attachment; IB, internal benefits; EB, external benefits; IRP, in-role performance; SI, sustained intention.

### 4.3 Exploratory factor analysis

Exploratory factor analysis (EFA) was conducted using the varimax method of principal component analysis with direct oblimin rotation on 12 items designed to measure volunteer motivation. The Kaiser–Meyer–Olkin (KMO) measure of sampling adequacy was 0.827, indicating that the correlation matrix was suitable for factor analysis. Additionally, Bartlett's test of sphericity was statistically significant (*χ*^2^ = 1691.406, *p* < .001), further confirming the appropriateness of the data for EFA (61). As shown in Table 2, the 12 items loaded onto four distinct factors with Eigenvalues greater than 1.0, collectively explaining 71.324% of the total variance.

**Table 2:** Results of EFA and reliability of each factor of volunteer motivation.

Items	Factor loading			
OA 1	.871	.159	.131	.114
OA 2	.853	.141	.131	.146
OA 3	.830	.118	.147	.213
VA 1	.071	.857	.155	.188
VA 2	.179	.838	.098	.103
VA 3	.166	.842	.211	.135
IB 1	.140	.131	.853	.111
IB 2	.168	.129	.793	.087
IB 3	.121	.154	.802	.193
EB 1	.197	.139	.112	.816
EB 2	.119	.166	.171	.833
EB 3	.171	.145	.153	.741
Reliability	.834	.954	.882	.791
Eigenvalue	5.131	1.231	1.416	1.222
Variance	40.67	9.76	11.22	9.68

The 12 items were retained as four distinct factors, each representing a dimension of sport volunteer motivation. Each factor comprised three items, with the identified factors being organization attachment (OA), volunteer attachment (VA), internal benefits (IB) and external benefits (EB). Specifically, the factor loadings were as follows: organization attachment (.830 to.871), volunteer attachment (.838 to.857), internal benefits (.793 to.853), and external benefits (.741 to.833).

The reliability of each factor was assessed using Cronbach's *α* analysis, with all coefficients exceeding the recommended. 70 threshold (69), ranging from .791 to .954. These results indicate that the items for each factor demonstrated strong internal consistency and reliability.

EFA was conducted on the six items designed to measure volunteer in-role performance (IRP) and sustained intention to volunteer (SI). The Kaiser–Meyer–Olkin (KMO) measure of sampling adequacy was 0.762, indicating that the data were suitable for factor analysis. Additionally, Bartlett's test of sphericity was statistically significant (*χ*^2^ = 629.741, *p* < .001), confirming that the correlation matrix was appropriate for EFA.

As shown in Table 3, the six items loaded onto two distinct factors with Eigenvalues greater than 1.0, collectively explaining 74.157% of the total variance**.** The three items measuring in-role performance had factor loadings ranging from .840 to .933, while the three items measuring sustained intention had factor loadings ranging from .733 to .881. Reliability analysis using Cronbach's α demonstrated strong internal consistency for both factors, with coefficients of .929 for in-role performance and .876 for sustained intention**,** both exceeding the .70 threshold for acceptable reliability. These results confirm the robustness and reliability of the measurement constructs.

**Table 3:** Results of EFA and reliability of in-role performance and sustained intention.

Items	Factor loading	
IRP 1	.933	.160
IRP 2	.883	.205
IRP 3	.840	.284
SI 1	.201	.881
SI 2	.219	.845
SI 3	.196	.733
Reliability	.929	.876
Eigenvalue	3.308	1.14
Variance	55.119	19.038

### 4.4 Influence of volunteer motivation on in-role performance

The results of the analysis on the impact of spectator motivation on in-role performance are presented in Table 4. The volunteer motivation factors explain 44.3% (*R*^2^ = .443) of the total variance, and the model was found to be a good fit (*F* = 41.25, *p* < .001). Among the volunteer motivation factors, organization attachment, volunteer attachment, and internal benefits were found to have a statistically significant effect on in-role performance. However, external benefits did not have a statistically significant impact on in-role performance.

**Table 4:** Results of multiple regression analysis for in-role performance.

Factor	β	SE	β	*t*	*p*
Constant	−.041	.340		−1.202	.023
OA	.027	.037	5.190***	5.190	.000
VA	.025	.042	4.399***	4.399	.000
IB	.131	.035	2.578*	2.578	.011
EB	.058	.041	.942	0.942	.347

*R* = .666, *R*^2^ = .443, Adjusted *R*^2^ = .432.

* *p* < .05.

*** *p* < .001.

### 4.5 Influence of volunteer motivation on sustained intention

The results of the analysis on the impact of volunteer motivation on sustained intention are presented in Table 5. The volunteer motivation factors explain 51.2% (*R*^2^ = .512) of the total variance, and the model was found to be a good fit (*F* = 54.51, *p* < .001). Among the volunteer motivation factors, volunteer attachment and internal benefits had a statistically significant effect on sustained intention to volunteer. However, organization attachment and external benefits did not have a statistically significant impact on sustained intention to volunteer.

**Table 5:** Results of multiple regression analysis for sustained intention.

Factor	β	SE	β	*t*	*p*
Constant	.331	.310		1.652	.099
OA	.042	.062	1.098	1.098	.273
VA	.459	.052	.299***	5.558	.000
IB	.275	.048	.188***	4.013	.000
EB	.078	.071	.041	.763	.446

*R* = .716, *R*^2^ = .512, Adjusted *R*^2^ = .503.

*** *p* < .001.

## 5 Discussion

The findings of this study underscore the nuanced role that different facets of volunteer motivation play in shaping both in-role performance and the intention to continue volunteering in adaptive sport events. Specifically, organization attachment (OA), volunteer attachment (VA), and internal benefits (IB) emerged as significant predictors of volunteer in-role performance, while external benefits (EB) did not exhibit a notable impact. These results align with existing literature suggesting that intrinsic and relational motivations are strong determinants of task-related outcomes (27, 32, 63). When volunteers feel emotionally connected to the organization and experience personal growth through their involvement, they are more inclined to invest effort in fulfilling their assigned responsibilities, whereas external benefits appear to be less crucial, particularly in adaptive sports settings.

A particularly noteworthy finding is the differential impact of volunteer attachment (VA) and internal benefits (IB) on sustained intention to volunteer, whereas organization attachment (OA) and external benefits (EB) did not show significant effects in this regard. These findings emphasize the need to prioritize intrinsically driven motives, such as self-actualization and altruistic fulfillment, rather than focusing on material or external rewards (8, 41). Furthermore, the prominence of volunteer attachment underscores the social and emotional aspects of volunteering. When individuals feel a personal commitment to the welfare of athletes and the surrounding community, they exhibit a stronger inclination to continue their involvement (23, 37).

The absence of a direct link between external benefits (EB) and either in-role performance or sustained intention resonates with research suggesting that extrinsic rewards, while helpful for initial recruitment, may not be sufficient for long-term engagement (18). Similarly, organization attachment (OA) did not significantly influence continued participation, indicating that while loyalty and pride in the organization can boost immediate performance, they may not necessarily translate into a strong incentive to volunteer again. This finding does not align with previous research. For example, Han et al. (41) identified organizational attachment as a strong predictor of volunteer engagement in the Olympics. It could be argued that in national-level mega sporting events, such as the Olympics or the FIFA World Cup, patriotism plays a crucial role in shaping organizational attachment, influencing volunteers' motivation to contribute to their country's success.

However, the results of this study suggest that the context of adaptive sport events may differ from other sporting events, as the observed patterns indicate distinct motivational dynamics. While the specific reasons for these differences remain unclear, it is likely that event-specific factors—such as the nature of the athlete-volunteer relationship and the community-based focus of adaptive sports—play a meaningful role. In this context, volunteers may be driven more by personal connections, empathy, and a sense of commitment to supporting athletes with disabilities, rather than by organizational loyalty. Although this assumption requires further empirical exploration, it aligns with the relational and inclusive nature often associated with adaptive sport settings. These insights reinforce the importance of considering event-specific motivational influences when designing volunteer recruitment and retention strategies.

These findings have significant practical implications for event managers and volunteer coordinators in the adaptive sports sector. Designing recruitment and retention strategies that prioritize internal benefits, particularly volunteer attachment as a key component of altruistic motivation identified in this study, may be more effective in fostering long-term volunteer commitment. Creating a supportive, community-oriented environment and emphasizing the importance of inclusion for athletes with disabilities can further enhance volunteer attachment. This, in turn, may lead to improved performance and sustained engagement, ensuring a more dedicated and motivated volunteer workforce in adaptive sports events.

However, there are several limitations to this study. First, the study had an imbalanced racial composition, with 96.7% of participants identifying as Asian-American. This demographic distribution reflects the festival's organization by the Korea ParaSports Association of USA and the predominant involvement of Korean-American athletes, staff, and volunteers. Future research could examine how volunteer motivations and experiences differ across diverse ethnic and cultural groups, particularly in sporting events designed for specific communities. Such research could yield valuable insights into the cultural factors that influence volunteer engagement, allowing organizations to develop more effective recruitment and retention strategies tailored to diverse populations. Moreover, a deeper understanding of these differences can help create more inclusive and supportive environments, ultimately enhancing the overall quality and impact of adaptive sports events.

Second, the study relied exclusively on self-reported survey data, which introduces the potential for social desirability bias and inaccurate self-assessment. Participants may have unintentionally overstated their intrinsic motivations or in-role performance to align with socially acceptable norms or perceived expectations. This limitation could impact the reliability and validity of the findings by obscuring the true relationships between motivational factors and volunteer outcomes.

Third, the cross-sectional design of this study limits the ability to establish causal relationships between motivational factors and volunteer outcomes. Because data were collected at a single point in time, it is not possible to determine the directionality of the observed relationships or assess how motivations may influence, or be influenced by, in-role performance and sustained intention over time. For example, while the findings suggest that internal benefits and volunteer attachment are associated with greater commitment, it remains unclear whether these motivations lead to continued volunteering or if prolonged engagement strengthens these motivations. Longitudinal studies would provide a more robust framework for examining how volunteer motivations and behaviors evolve, particularly across multiple events, varying organizational contexts, or different phases of a volunteer's lifecycle. Such designs could offer deeper insights into the dynamic nature of volunteer engagement and inform more effective, time-sensitive recruitment and retention strategies.

Lastly, the study focused on a single event context, which may limit the generalizability of the findings to other adaptive sport events with different organizational structures, cultural settings, or participant demographics. Future studies could compare findings across various adaptive sport contexts to better understand how event-specific factors influence volunteer motivation and engagement.

In addition, future research could expand on this work by exploring additional motivational dimensions, such as personality traits or role identity, and by employing longitudinal designs to track changes in volunteers' motivations and commitment over time. Comparative studies across different types of adaptive sports or cultural settings may also provide deeper insights into how diverse volunteer populations experience and respond to motivational influences. Furthermore, mixed-methods approaches—combining quantitative surveys with qualitative interviews—could enrich our understanding of the lived experiences of volunteers, ultimately helping to refine and enhance volunteer management strategies in adaptive sports and beyond.

## 6 Conclusion

In sum, this study significantly advances the understanding of how various dimensions of volunteer motivation influence both in-role performance and the sustained intention to volunteer in adaptive sport events. The findings underscore the pivotal role of volunteer attachment (VA) and internal benefits (IB) as key drivers of not only immediate volunteer effectiveness but also long-term commitment. In contrast, external benefits (EB) had no meaningful impact on either outcome, and organization attachment (OA) influenced only short-term performance, not continued involvement.

These insights carry important implications for adaptive sport organizations aiming to build and retain a strong volunteer base. Efforts should prioritize cultivating meaningful, emotionally resonant connections between volunteers, athletes, and the broader community. Equally important is the creation of environments that support volunteers’ personal growth, fulfillment, and sense of purpose. By focusing on these intrinsic and relational motivators, organizations can foster a more committed, high-performing, and resilient volunteer workforce—ultimately enhancing the quality and sustainability of adaptive sport events.

## Data Availability

The raw data supporting the conclusions of this article will be made available by the authors, without undue reservation.

## Footnotes

Strigas A. *The assessment of motives and the development of a typology of motivational factors for volunteers in marathon running events* (Unpublished Doctoral Dissertation). Florida State University, Tallahassee, FL, United States (2001). Available at: https://www.proquest.com/docview/304692881?pq-=&utm_source=chatgpt.com&sourcetype=Dissertations%20&%20Theses
